# The Cancer Dependency Map enables drug mechanism‐of‐action investigations

**DOI:** 10.15252/msb.20209757

**Published:** 2020-07-21

**Authors:** Francisca Vazquez, Jesse S Boehm

**Affiliations:** ^1^ Broad Institute of Harvard and MIT Cambridge MA USA

**Keywords:** Chemical Biology

## Abstract

How do small molecules exert their effects in mammalian cells? This seemingly simple question continues to represent one of the fundamental challenges of modern translational science and as such has long been the subject of intense scientific scrutiny. In their recent study, Garnett and colleagues (Gonçalves *et al*, 2020) demonstrate proof‐of‐concept for a new way to attack this problem systematically for Oncology drugs, by identifying correlated CRISPR‐ and drug‐killing profiles in the Cancer Dependency Map dataset.

## Graphical abstract

Deciphering the mechanism by which small molecules act in human cells is challenging. In their recent study, Garnett and colleagues present an approach to tackle this problem by correlating CRISPR‐drug‐killing profiles in the Cancer Dependency Map.

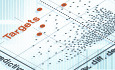

Deciphering the mechanism(s)‐of‐action (MoA) by which small molecules act in human cells is key to identifying why patients do or do not respond to treatment, for developing next‐generation molecules with improved efficacy and selectivity and for identifying and preempting mechanisms of resistance. Yet, doing so is highly challenging.

For small molecules emerging from target‐based discovery campaigns, it is assumed that the answer revolves around the biochemical perturbation of the target. Despite this assumption, polypharmacological effects may play significant roles. Reciprocally, small molecules that emerge from phenotypic cellular‐based screens have historically required laborious biophysical approaches to unmask their MoA. A highly cited example is the elegant discovery of the MoA of the natural product rapamycin: the FKBP12 protein that disrupts mTOR signaling (Brown *et al*, 1994).

In the case of cancer, several promising new genomic frontiers are now emerging that are beginning to accelerate progress in MoA. First, the use of genetic RNAi or CRISPR modifier screens to identify rescue or sensitization to anti‐cancer drug killing has been a powerful approach (Jost & Weissman, 2018; Colic *et al*, 2019). Second, the use of gene expression and/or high content imaging as a surrogate measurement has enabled the assessment of “connectivity” in signature space (e.g., between a known perturbation and that of a small molecule) (Subramanian *et al*, 2017). Despite these advances, such approaches are typically limited to specific cancer contexts.

But can the power of CRISPR be leveraged to resolve the MoA of a small molecule for cancer *systematically* across a wide diversity of cellular contexts? This question is the focus of Gonçalves *et al*, reported in the current issue of *Molecular Systems Biology*. The authors hypothesize that a small molecule and a CRISPR genetic knockout that exert the same pattern of killing across cancer cell line models are likely to function through similar mechanisms.

To address this hypothesis, the authors leverage recently emerging data from the Cancer Dependency Map (depmap.org; depmap.sanger.ac.uk) in which hundreds of molecularly characterized cancer cell line models have been similarly subjected to genome‐wide RNAi or CRISPR (Tsherniak *et al*, 2017; Behan *et al*, 2019) and pharmacological (Iorio *et al*, 2016) profiling. These data have recently been found to be highly reproducible across institutions, suggesting an opportunity for integration to increase power.

The premise in this proof‐of‐concept manuscript that focuses on established cancer drugs with largely known MoAs is that the correlation in viability between one of ∼17,000 genetic knockouts and one of 397 established drugs across 484 diverse cell lines should rediscover the MoA. While examples of success have been reported, a broad‐scale study of this new use of the Cancer Dependency Map data has only become possible recently.

Through an extensive series of supervised linear regression analyses, they demonstrate the merits of this approach. They find that in 26% of cases, the killing pattern of the drug is directly phenocopied by the CRISPR killing pattern of the known drug target. They investigate the 264 cases in which this is not the case and find, using protein–protein interactions, 76 additional examples in which the small molecule's killing pattern correlates with a first, second, or third order interactor. Thus, in aggregate, the authors conclude that either the target or pathway can be rediscovered in roughly 48% of cases.

The authors next investigate some interesting examples. For instance, in the example of isoform‐specific PI3K inhibitors, the expected genetic knockdowns indeed correlate. In the case of MCL1 inhibition, they discover an exciting relationship between the MARCH5 E3 ligase and MCL1 inhibition. This finding has now been confirmed by other groups including reports of the MARCH5‐dependent degradation of the MCL1/NOXA complex. This discovery should prove very interesting for the therapeutic exploitation of the MCL1 dependency in many human cancers.

Finally, the authors explore concordant biomarkers that explain the sensitivity of both genetic and small molecule perturbations. They show how these biomarkers can inform MoA, along the lines of previous work focused on small molecules (Rees *et al*, 2015). For instance, the authors identify tumor necrosis alpha expression as a robust biomarker of sensitivity to both cIAP inhibitors as well as genetic knockout of members of the cIAP pathway.

This manuscript shares some similarities to a recent report (Corsello *et al*, 2020) that together lay out the exciting potential to resolve the MoA of small molecules in a new way, using the Cancer Dependency Map (Fig 1). One limitation in the current report is the focus on established anti‐cancer drugs, which have potent efficacy and (typically) highly refined MoA. Thus, while this is a useful proof‐of‐concept, it is unclear ultimately how the approach will work where the real MoA challenge is, which is for compounds in development, in which the drug‐killing effect is weaker and often less specific.

**Figure 1:**
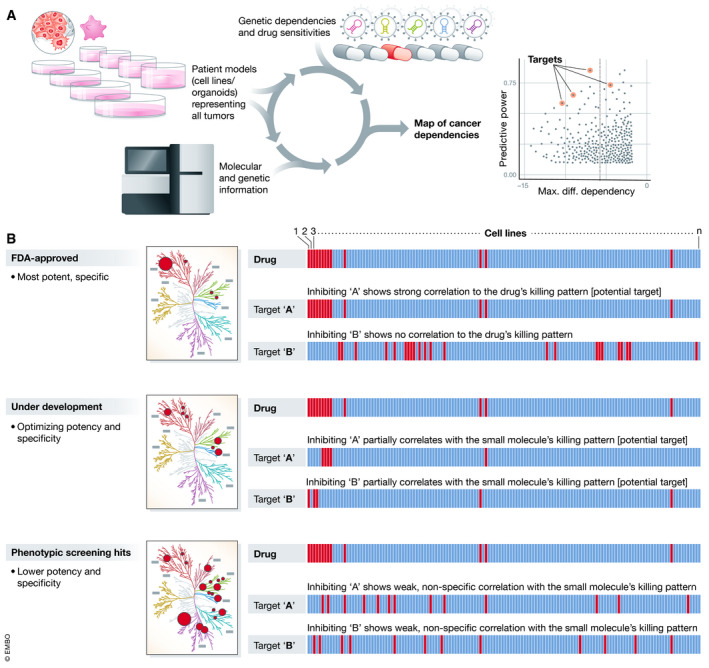
Using the Cancer Dependency Map data to generate MoA hypotheses (A) By profiling hundreds of patient models of human cancer, the Cancer Dependency Map systematically identifies gene dependencies, small molecule sensitivities, and the markers that predict their response. (B) Correlations of genetic dependencies and drug sensitivities across cell lines can inform small molecule target(s) identification and mechanism‐of‐action. Red bars: cell killing; blue bars: no cell killing.

Additionally, the regression methods used in this paper may not fully account for the notion that most small molecules have diverse polypharmacological effects that together may conspire to explain the mechanism of killing across various cellular contexts. As the scale of the Dependency Map dataset grows, future computational approaches may be leveraged to produce consensus matches that aggregate individual targets in more complex ways.

With these caveats aside, this report provides new insight into how valuable the Cancer Dependency Map is likely to be as a reference to guide MoA studies throughout the drug discovery process (Fig 1). In the years ahead, it will be important for the field to aspire to learn generalizable lessons around the strengths and weaknesses of various experimental and computational modalities to finally solve this important challenge for oncology once and for all.
